# Childhood pneumothorax in Birt‐Hogg‐Dubé syndrome: A cohort study and review of the literature

**DOI:** 10.1002/mgg3.373

**Published:** 2018-02-13

**Authors:** Marianne Geilswijk, Elisabeth Bendstrup, Mia Gebauer Madsen, Mette Sommerlund, Anne‐Bine Skytte

**Affiliations:** ^1^ Department of Clinical Genetics Aarhus University Hospital Aarhus N Denmark; ^2^ Department of Respiratory Disease and Allergy Aarhus University Hospital Aarhus C Denmark; ^3^ Department of Urology Aarhus University Hospital Aarhus N Denmark; ^4^ Department of Dermatology Aarhus University Hospital Aarhus C Denmark

**Keywords:** Birt‐Hogg‐Dubé syndrome, pediatrics, renal cell carcinoma, spontaneous pneumothorax

## Abstract

**Background:**

Birt‐Hogg‐Dubé syndrome (BHD) is an autosomal dominantly inherited cancer predisposition syndrome associated with an increased risk of spontaneous pneumothorax (SP) and renal cell carcinoma in the adult population. Recent studies suggest that BHD accounts for up to 10% of all SP in adults and BHD in children with SP have been reported.

**Methods:**

To explore to what extent BHD is the cause of childhood pneumothorax, we studied a Danish BHD cohort consisting of 109 cases from 22 families. Clinical data was gathered by review of medical records. A systematic literature search concerning childhood and adolescence pneumothorax in BHD was performed and identified publications reviewed.

**Results:**

In our cohort, three of 109 BHD cases experienced childhood pneumothorax, corresponding to a prevalence of 3%. Reviewing the literature, data regarding more than 800 BHD cases were covered. Only seven previously published cases of childhood pneumothorax in BHD were identified.

**Conclusion:**

Our findings suggest that BHD is likely the cause of a larger subset of childhood pneumothoraces than hitherto recognized. Awareness of BHD as a cause of childhood pneumothorax needs to be raised to provide patients and relatives with the possibility of specialized management of SP and regular renal cancer surveillance.

## 1 INTRODUCTION

### 1.1 Background

Birt‐Hogg‐Dubé syndrome (BHD, OMIM#135150) is a rare autosomal dominantly inherited cancer predisposition syndrome, first described in 1977 by the Canadian physicians Birt, Hogg, and Dubé (Birt, Hogg, & Dube, 1977). In 2002, it was recognized that BHD is caused by germline pathogenic variants in the folliculin gene (*FLCN*). The exact prevalence of BHD is not known, but so far more than 600 families are reported worldwide (BHD foundation, 2017). Classic manifestations typically occur after the age of 20 years and include benign skin tumors (fibrofolliculomas, trichodiscomas, and acrochordons), an increased risk of benign and malignant kidney tumors as well as basal lung cysts and recurrent spontaneous pneumothoraces (SP) (Schmidt & Linehan, 2015). The BHD diagnosis is based on phenotypical findings, family history, and/or the detection of a germline pathogenic variant in *FLCN* (Menko et al., 2009). To date more than 150 different pathogenic *FLCN* variants have been reported and at molecular genetic testing of individuals with clinical BHD, the diagnostic yield is >80% (Houweling et al., 2011). Genotype–phenotype correlations have been proposed but not clearly established. The gene product, folliculin, serves multiple roles in cellular homeostasis which are not all fully elucidated. It is well‐established that folliculin interacts with the AKT‐mTOR pathway as a tumor suppressor, but the protein has also been shown to be involved in cell adhesion, mitochondrial metabolism, autophagy, and ciliogenesis (Hartman et al., 2009; Schmidt & Linehan, 2015).

Due to incomplete penetrance of clinical manifestations and marked variability in expression, BHD is generally assumed to be underdiagnosed. Symptomatic as well as nonsymptomatic individuals with BHD are advised regular renal cancer surveillance from age 20 years and specialized management of SP in order to reduce recurrence rate (Jensen et al., 2017; Menko et al., 2009).

### 1.2 Clinical manifestations

Fibrofolliculomas are benign skin tumors arising from the hair follicles, highly suggestive of BHD and in some cohorts reported in up to 70%–85% of cases (Toro et al., 2008). They do not undergo malignant transformation but localized in the face, neck, and upper trunk fibrofolliculomas can cause cosmetic discomfort.

Renal tumors in BHD are often bilateral and multifocal. In a recent review, the risk of renal cell carcinoma (RCC) in BHD was estimated to be 16% at age 70 years (Houweling et al., 2011). Mean age of RCC diagnosis is 46 years in BHD but ranges 14–83 years. Often RCC in BHD follows an indolent course but aggressive and metastasizing disease is also reported (Houweling et al., 2011). In Denmark, the recommendations from the BHD Consortium are applied and affected individuals are offered renal imaging surveillance every second year to reduce the risk of RCC (Menko et al., 2009).

Lung involvement in BHD in the form of bilateral, basal, subpleural lung cysts are seen in computed tomography (CT) of the chest in more than 80% of the examined cases (Toro et al., 2007). The role of *FLCN* haploinsufficiency in the development of cystic lung disease is not fully elucidated. It has been proposed that the cysts in BHD are the result of diminished stretch tolerance in the alveolar wall, as studies have shown that folliculin deficiency increases cell–cell adhesion in human bronchial epithelial cells (Kennedy, Khabibullin, & Henske, 2016; Khabibullin et al., 2014). The stretch‐hypothesis is a plausible explanation for the fact that the BHD cysts are localized in the basal compartments of the lungs rather than in the apices (Kennedy et al., 2016). BHD lung cysts are thin‐walled and irregular in shape and size and can be radiologically distinguished from other cystic lung diseases (Gupta, Sunwoo, & Kotloff, 2016; Tobino et al., 2011). Furthermore, the cysts in BHD do not progress and lung function is normal (Johannesma, Houweling, et al., 2014). The presence of BHD lung cysts is positively correlated with the well‐established increased risk of recurrent SP. Thus, the age‐adjusted risk of SP in BHD is 50 times higher than in the background population and recurrence rates are high (Zbar et al., 2002). Mean age of onset is 38 years and the majority of BHD cases experiencing SP will suffer repeated and bilateral incidences (Kunogi et al., 2010; Toro et al., 2007). Symptoms and handling of SP in BHD do not differ from that of SP of other causes. Because of the significant recurrence risk, some clinicians recommend pleurodesis to be considered at an early state (Gupta et al., 2016). In line with the interfamilial variability seen in BHD it is well recognized that BHD families exist in which SP is the sole manifestation (Gunji et al., 2007; Painter, Tapanainen, Somer, Tukiainen, & Aittomaki, 2005). Based on molecular genetic screening studies of persons with primary SP, it has been suggested that BHD accounts for up to 10% of all SP in the adult population (Johannesma et al., 2015; Ren et al., 2008; Zhang et al., 2016). There are only few previous reports of pediatric pneumothorax in BHD and similar studies in pediatric populations have not been published. We present a cohort study and a review of the literature and hypothesize that BHD accounts for a significant part of childhood pneumothorax cases.

## 2 METHODS AND MATERIAL

### 2.1 Cohort study

With approval by the Danish Data Protection Agency (ref no. 1‐16‐02‐626‐16)) a database was established to characterize the phenotypical spectrum of BHD in the Central Denmark Region (CDR). Families included in the database were recruited from the Dept. of Clinical Genetics, Aarhus University Hospital, where to index persons had been referred from the Dept. of Respiratory Disease (six of 22 families), the Dept. of Dermatology (three of 22) or the Dept. of Urology (four of 22), all Aarhus University Hospital, or from general practitioners and other specialties (nine of 22). All individuals included in the database are confirmed carriers of a pathogenic *FLCN* variant, either by direct molecular genetic testing or as obligate carriers. Medical records have been reviewed and relevant clinical data gathered. For this, written informed consent was obtained from all individuals who were alive at the time of inclusion. In accordance with Danish legislation, medical records regarding persons who had passed away at the time of inclusion have been reviewed without the consent from relatives.

### 2.2 Review of the literature

A PubMed search with the term “Birt‐Hogg‐Dubé Syndrome” and the Medical Subject Headings (MeSH) term [pneumothorax] was performed on 9 June 2017. An Embase search with the terms “Pneumothorax” and “Birt‐Hogg‐Dubé syndrome” was performed on 12 June 2017. Three PubMed searches with the term “Birt‐Hogg‐Dubé syndrome” and MeSH terms [lung], [child], and [adolescence], respectively, as well as four PubMed searches with the term “*FLCN*” and MeSH terms [pneumothorax], [lung], [child], and [adolescence], respectively, were performed on 22 June 2017. Three Embase searches with the term “Birt‐Hogg‐Dubé syndrome” and the terms “lung,” “child,” and “adolescent,” respectively, as well as four Embase searches with the term “*FLCN*” and the terms “pneumothorax,” “lung,” “child,” and “adolescent,” respectively, were performed on 23 June 2017. Articles were restricted to those published in English language. Conference abstracts were not included. Cross‐referencing was done to avoid duplicates. An additional nine reports were identified from reference lists. A total of 287 articles were identified for review. Two cases were reported twice/three times respectively (Bessis et al. 2006; Kluger et al. 2010; Gunji et al. 2007; Kunogi et al. 2010; Tobino et al., 2011).

## 3 RESULTS

In the following, “childhood” is defined as ≤ 15 years of age and “adolescence” as 16 ≤ 20 years of age.

### 3.1 Cohort study

Twenty‐two families with a minimum of three generations, corresponding to 109 individual BHD cases have been included in the database. Of these, 92 cases are currently alive. The data collected spans more than a century, the earliest and most recent years of birth registered being 1892 and 2001, respectively. Fifty‐two (48%) of the included cases are males and 57 (52%) females. Mean age at BHD diagnosis is 53 years and ranges 15–95 years. Forty‐three of 109 cases have a verified history of SP, corresponding to 39% (Table 1). In this subgroup, 40% are male and 60% female. Recurrent pneumothoraces are reported in 40% of the pneumothorax cases. Age of first pneumothorax is known in 30 of the 43 cases. The majority (43%), developed their first pneumothorax in the fourth decade, reflected in a mean age of onset of 35 years. Four of the 43 cases developed pneumothorax before age 20 years and of these four, three experienced their first event at ages 13, 14, and 14 years. Thus, 7% of the BHD cases with a history of SP experienced their first episode in childhood. The three cases of childhood pneumothorax occurred in 1985, 1987, and 2015 in otherwise healthy boys, who all had a family history of pneumothorax. Two of the cases belong to the same family. Index persons in the two families were referred for clinical genetic evaluation due to (1) a family history of SP and (2) suspected Lynch syndrome (i.e., an inherited risk of colorectal and other cancers), respectively.

**Table 1:** Spontaneous pneumothorax in the Central Denmark Region BHD cohort

Age interval (years)	SP^a^ cases (number)	Percentage of SP subgroup (n = 43)	Percentage of whole cohort (n = 109)
≤ 10	0	0	0
11–15	3	7%	3%
16–20	1	2%	1%
21–30	5	12%	5%
31–40	14	33%	13%
41–50	5	12%	5%
51–60	0	0	0
61–70	1	2%	1%
≥ 71	1	2%	1%
Age unknown	13	30%	12%
Total	43	100%	39%

a Spontaneous pneumothorax.

### 3.2 Review of the literature

The 287 articles reviewed contain a total of 33 cases of childhood/adolescence pneumothorax in BHD (Bessis et al., 2006; César, Baudrier, Mota, & Azevedo, 2016; Chung, Ramos‐Caro, Beers, Ford, & Flowers, 1996; Demir & Cobanoglu, 2016; Ding et al., 2015; Furuya et al., 2016; Gunji et al., 2007; Gupta et al., 2017; Houweling et al., 2011; Johannesma, van den Borne, et al., 2014; Johannesma et al.,^2015^; Kolb, King, & Pearse, 2013; Kunogi et al., 2010; Li, Ning, He, & Gong, 2017; Maffe et al., 2011; Monserrate, Al‐Jaghbeer, & Cirino Marcano, 2017; Predina, Kotloff, Miller, & Singhal, 2011; So, 2009; Toro et al., 1999). Twelve of these 33 cases are described in case reports and the remaining 21 cases are reported as parts of larger cohort studies. Of the 33 identified cases, seven concern children at or below age 15 years. Data regarding gender and family history is available in five of the seven childhood cases. All were males, and four had a positive family history of either fibrofolliculomas (three) or SP (one). None of the childhood cases had any other clinical manifestations of BHD at the time of their first SP. Further details regarding age, clinical manifestations of BHD, genotype, and family history of all identified cases of childhood/adolescence SP in BHD are specified in Table 2.

**Table 2:** Childhood/adolescence spontaneous pneumothorax in BHD, identified in the literature

Author (Year)	Article type	Age^a^	Gender	Other manifestations^b^	*FLCN* variant	Family history
Chung et al. (1996)	Case report	15	M	FF(20)	–	FF
Toro et al. (1999)	Cohort	≤ 20	F	FF	–	FF, RCC
Bessis et al. (2006)	Case report	7	M	None	c.458delG	FF
Gunji et al. (2007)	Cohort	16	F	None	c.1285dupC	None
So (2009)	Case report	17	M	N/A	Unknown	FF, SP
Kunogi et al. (2010)	Cohort	16	F	None	c.1539‐? c.1740 + ?del	SP
		20	F	FF	c.1347_1353dupCCACCCT	SP
Predina et al. (2011)	Case report	18	M	N/A	Unknown	FF, SP, RCC
		17	M	N/A	Unknown	FF, SP, RCC
Maffe et al. (2011)	Cohort	20	F	RCC(34)	c.1285dupC	None
Houweling et al. (2011)	Cohort	18	F	N/A	c.610_611delinsTA	FF, SP, RCC
Kolb et al. (2013)	Case report	19	F	FF	c.453delG	SP, RCC
Johannesma (2014)	Case report	14	M	None	c.1177‐5_1177‐3delCTC	None
		14	M	None	c.1301‐7_1304del;1323delCinsGA	FF
Johannesma et al. (2015)	Cohort	20	F	FF	c.610_611delinsTA	N/A
Ding et al. (2015)	Cohort	18	M	None	c.872‐429_1740 + 1763del	SP
Demir and Cobanoglu (2016)	Case report	10	M	None	c.499C>T	SP
Furuya et al. (2016)^c^	Cohort	15	N/A	N/A	Yes, unspecified	N/A
		19	N/A	N/A	Yes, unspecified	N/A
		18	N/A	N/A	Yes, unspecified	N/A
		20	N/A	N/A	Yes, unspecified	N/A
		19	N/A	N/A	Yes, unspecified	N/A
		20	N/A	N/A	Yes, unspecified	N/A
		20	N/A	N/A	Yes, unspecified	N/A
		20	N/A	N/A	Yes, unspecified	N/A
		20	N/A	N/A	Yes, unspecified	N/A
César et al. (2016)	Case report	19	M	FF	c.50G>C	RCC
Li et al. (2017)	Case report	17	M	N/A	c.946_947delAG	SP, RCC
Monserrate et al. (2017)	Case report	17	M	N/A	Yes, unspecified	SP
Gupta et al. (2017)	Cohort	14	N/A	N/A	Unknown	N/A
		≤ 20	N/A	N/A	Unknown	N/A
		≤ 20	N/A	N/A	Unknown	N/A
		≤ 20	N/A	N/A	Unknown	N/A

FF, Fibrofolliculomas; N/A, Data not available; RCC, Renal cell carcinoma; SP, Spontaneous pneumothorax.

a Age at pneumothorax.

b Fibrofolliculomas and/or renal cell carcinoma. Age of onset in parentheses.

c Ages specified by Furuya, personal communication.

## 4 DISCUSSION

It is well known, that in general the risk for SP is increased in individuals with a positive family history of SP and also that pathogenic variants in *FLCN* are inherited in families in which SP is the sole manifestation of BHD (Gunji et al., 2007; Toro et al., 2008). The same is true for fibrofolliculomas in families with only cutaneous findings of BHD. Thus, it would be reasonable to expect the prevalence of childhood pneumothorax to be higher in populations ascertained because of pulmonary findings than in populations with mainly skin manifestations. During our literature review, we did not find clear evidence for this. In their studies altogether, Zbar, Schmidt, and Toro surveyed 145 BHD families of which 132 were recruited from dermatologic clinics and none solely because of SP (Schmidt et al., 2005; Toro et al., 2008; Zbar et al., 2002). In these studies, the overall prevalence of SP ranged 22.5%–38%. To the best of our belief, data concerning pulmonary manifestations in these populations, including age of first pneumothorax, have been published elsewhere without the notion of cases of childhood/adolescence pneumothorax (Toro et al., 2007). In 2011, Houweling reported on clinical manifestations in 53 BHD families of which 48 were referred by dermatologists. In this population, the overall prevalence of SP was estimated to be 24%. Houweling reported one case of adolescence pneumothorax. Contrary to these studies, the majority of index cases studied by Furuya et al. were evaluated for BHD after one or recurrent episodes of SP (Furuya et al., 2016). In their cohort, consisting of 312 BHD cases, the overall prevalence of SP was 70%. One case of childhood pneumothorax and eight cases of pneumothoraces in adolescence were described. Likewise, Gupta et al. described 104 BHD cases recruited because of pulmonary findings. In this cohort 76% experienced one or recurrent pneumothoraces (Gupta et al., 2017). Data are not specified but four cases of childhood/adolescence pneumothorax is reported, the youngest being 14 years old.

To the best of our knowledge, we have reviewed all available English‐language publications regarding SP in BHD, and we have covered published data regarding more than 800 BHD cases ascertained for various reasons. In the literature, we encountered seven previous reports of SP in BHD at or below age 15 years. None of the large cohort studies describe more than one case of childhood pneumothorax each, regardless of potential selection bias (Li et al., 2017; Maffe et al., 2011). Only in the case series by Johannesma et al. addressing specifically childhood pneumothorax in BHD, two incidences are reported (Johannesma, van den Borne, et al., 2014).

We find that the BHD population in our cohort is comparable to those described by other investigators in terms of number of persons included as well as age and gender distribution (Houweling et al., 2011; Toro et al., 2008; Zbar et al., 2002). Based on the referral history of the included families, we find it unlikely that our cohort is biased toward families mainly presenting lung manifestations. Among our BHD cases, we found an overall SP prevalence of 39%. This is slightly higher than that reported by other investigators but not at all in the level reported by Furuya and Gupta (Furuya et al., 2016; Gupta et al., 2017; Kluger et al., 2010; Schmidt et al., 2005; Toro et al., 2007; Zbar et al., 2002). In this setting, it is remarkable that we in our cohort of 109 BHD cases identified three cases of childhood pneumothorax. So far, it seems that BHD in childhood pneumothorax has been underreported or even underdiagnosed. In the previously published BHD cohort studies, each family consists of two BHD cases on average. To compare, the families included in our cohort comprise an average of five BHD cases. It is possible that extended family investigation in our cohort partly explains the observed difference in prevalence of childhood SP compared to previous findings.

Another contributory cause for the low number of previously reported cases of BHD in childhood pneumothorax is very likely unawareness of the syndrome and its potential clinical manifestations in childhood. We find it probable that a significant amount of childhood pneumothoraces are due to BHD and wrongly have been ascribed to chance, male sex, body habitus, or a nonspecified familial predisposition to SP. Compared to other inherited conditions with an increased risk of SP in childhood, like Marfan syndrome and Ehlers‐Danlos syndrome, BHD is not easily recognized in a child unless relevant questions upon family history are asked. Children suffering from SP are not routinely subjected to chest CT, which means that any subpleural basal lung cysts indicating the diagnosis are not disclosed. We do not opt for chest CT as a standard diagnostic tool in childhood or adolescent pneumothorax, but instead awareness of BHD must be raised in relevant specialties.

Our findings suggest that BHD is the underlying cause of an even larger subset of childhood pneumothoraces than hitherto recognized. Diagnosing BHD families is important not merely for the symptomatic individual but also for the possible nonsymptomatic carriers who should also be offered renal cancer surveillance in adulthood as well as specialized management in the case of SP. Future studies aimed at determining the prevalence of BHD in a childhood pneumothorax cohort are warranted.

## CONFLICT OF INTEREST

The authors declare no conflicts of interest regarding publishing the manuscript entitled “Childhood pneumothorax in Birt‐Hogg‐Dubé syndrome: A cohort study and review of the literature” in Molecular Genetics & Genomic Medicine.
